# Persistence of Ebola virus in various body fluids during convalescence: evidence and implications for disease transmission and control

**DOI:** 10.1017/S0950268816000054

**Published:** 2016-01-25

**Authors:** A. A. CHUGHTAI, M. BARNES, C. R. MACINTYRE

**Affiliations:** School of Public Health and Community Medicine, Faculty of Medicine, University of New South Wales, Sydney, Australia

**Keywords:** Body fluids, Ebola virus, transmission

## Abstract

The aim of this study was to review the current evidence regarding the persistence of Ebola virus (EBOV) in various body fluids during convalescence and discuss its implication on disease transmission and control. We conducted a systematic review and searched articles from Medline and EMBASE using key words. We included studies that examined the persistence of EBOV in various body fluids during the convalescent phase. Twelve studies examined the persistence of EBOV in body fluids, with around 800 specimens tested in total. Available evidence suggests that EBOV can persist in some body fluids after clinical recovery and clearance of virus from the blood. EBOV has been isolated from semen, aqueous humor, urine and breast milk 82, 63, 26 and 15 days after onset of illness, respectively. Viral RNA has been detectable in semen (day 272), aqueous humor (day 63), sweat (day 40), urine (day 30), vaginal secretions (day 33), conjunctival fluid (day 22), faeces (day 19) and breast milk (day 17). Given high case fatality and uncertainties around the transmission characteristics, patients should be considered potentially infectious for a period of time after immediate clinical recovery. Patients and their immediate contacts should be informed about these risks. Convalescent patients may need to abstain from sex for at least 9 months or should use condoms until their semen tests are negative. Breastfeeding should be avoided during the convalescent phase. There is a need for more research on persistence, and a uniform approach to infection control guidelines in convalescence.

## INTRODUCTION

The recent Ebola virus (EBOV) outbreak in West Africa spread to ten countries, with more than 28 600 cases and 11 300 deaths reported by the World Health Organization (WHO) [1]. Although, the outbreak is over, Ebola is an understudied infection and many aspects of viral transmission remain unclear. Direct contact with blood and body fluids is considered the primary transmission mode [2], but other modes may be possible [3]. Current evidence suggests that EBOV may persist in the some body fluids after clinical recovery and clearance of the virus from blood [4–8], although limited data are available [9]. The aim of this study was to review the current evidence around the persistence of EBOV in various body fluids during convalescence and to discuss its implication on disease transmission and control.

## METHODS

We conducted a systematic review. We searched articles from Medline (January 1976 to October 2015) and EMBASE (1988 to October 2015). The following key words were included for the search: ‘Ebola virus and blood/body fluids’, ‘Ebola and convalescence’, ‘Ebola and case report’, ‘Ebola and laboratory test’, ‘Ebola and urine’, ‘Ebola and sweat/skin’, ‘Ebola and semen’, ‘Ebola and saliva’, ‘Ebola and breast milk’, ‘Ebola and conjunctiva/eye’, ‘Ebola and faeces’, ‘Ebola and vagina’, ‘Ebola and vomit’, ‘Ebola and sputum’ and ‘Ebola and secretions’. One author (M.B.) conducted the initial search and reviewed the titles and abstracts of the studies to prepare an initial list of papers. All authors (M.B., R.M., A.A.C.) independently reviewed the full text of these paper and selected final papers to be included in the review (see Table 1). Studies published in English language were included. As the aim was to examine the persistence of EBOV in various body fluids after the blood samples were negative for EBOV (i.e. during convalescence), we excluded studies which confirmed the presence of EBOV in blood only. Presence of EBOV in blood pertained to acute infection, not convalescence.

**Table 1.** Summary of studies which tested body fluids in convalescent Ebola patients – proportion of positive samples and last day of positive sample*

Study and type of Ebola strain	Urine		Sweat/skin		Semen		Saliva		Breast milk		Faeces		Conjunctiva		Vagina		Other†		
	Culture	PCR or ELISA	Culture	PCR or ELISA	Culture	PCR or ELISA	Culture	PCR or ELISA	Culture	PCR or ELISA	Culture	PCR or ELISA	Culture	PCR or ELISA	Culture	PCR or ELISA	Culture	PCR or ELISA	Remarks
Emond *et al*. 1977 [6] (Sudan strain)	0/5 (N)				2/4 (61)						0/2 (N)						0/2 (N)		Other – throat swab negative. Last blood culture negative on day 9.
Rodriguez *et al.* 1999 [4] (Zaire strain)	0/19 (N)	0/19 (N)	0/19 (N)	0/19 (N)	1/10 (82)	7/10 (101)	0/19 (N)	0/19 (N)			0/18 (N)	2/18 (29)	0/20 (N)	1/20 (22)	0/15 (N)	3/15 (33)			RT–PCR used. Blood samples taken for 13 patients, at days 30, 33 and 62, respectively. Both PCR and culture positive for semen, but only PCR positive for faeces, conjunctiva and vagina.
Rowe *et al*. 1999 [7] (Zaire strain)	0/95 (N)	0/95 (N)	0/84 (N)	0/84 (N)	0/11 (N)	6/11 (91)	0/86 (N)	0/86 (N)			0/79 (N)	0/79 (N)	0/85 (N)	0/85 (N)	0/44 (N)	0/44 (N)			ELISA used to test presence of EBO antigen and IgM and IgG antibodies. RT–PCR used for semen samples only and was positive. Culture was negative.
Richards *et al.* 2000 [10] (Zaire strain)		1/NA (23)			1/1 (19)														Blood culture negative on day 12 and antigen test negative on day 20. ELISA used for IgM antibodies and virus antigen detection. Total number of urine samples not recorded.
Bausch *et al*. 2007 [8] (Sudan strain)		0/4 (N)		0/3 (N)	1/2 (45)	1/2 (45)		0/4 (N)	1/1 (15)	1/1 (15)								0/2 (N)	Other – one sample each from vomit and sputum. RT–PCR used. Only convalescent phase results presented. Both culture and PCR were positive.
Kreuels *et al.* 2014 [5] (Zaire strain)	1/NA (26)	1/NA (30)	1/NA (N)	1/NA (40)				1/NA (N)				1/NA (N)		1/NA (N)				1/NA (N)	Other – sputum sample negative. Blood culture negative on day 14 and antigen test (RT–PCR) negative at day 17. Total number samples not recorded. Both PCR and culture positive on urine while only PCR positive on sweat.
Lyon 2014 [11] (Zaire strain)		1/NA (28)																	RT–PCR used. Viral RNA detected in plasma and urine by RT–PCR 28 days after onset of illness.
Moreau *et al*. 2014 [13] (Zaire strain)		1/1 16								0/1 (15)									RT–PCR used. Last blood sample negative for virus on day 14.
Baggi *et al*. 2014 [14] (Zaire strain)																		8/8 22	Blood was negative by PCR on day 17 of illness (day 10 of admission). Positive in amniotic fluid, placenta, fetal blood and fetal meconium.
Christie *et al*. 2015 [15] (Zaire strain)					0/1	1/1 199													RT–PCR used. PCR positive but culture negative.
Varkey *et al*. 2015 [17] (Zaire strain)																	1/1 (63)	1/1 (63)	RT–PCR used, 1 sample from aqueous humor. Both PCR and culture positive.
Deen *et al*. [19] (Zaire strain)						46/93 272													RT–PCR. Culture results still to come

N, Sample result is negative; NA, number of total samples unknown.

* Each cell provides number of positive samples/number of total sample collected (and last day of positive sample by body fluid/site).

† Other includes negative samples from throat swab, vomit and sputum.

Grey literature sources included the websites of the WHO and Centers for Disease Control and Prevention (CDC) as well as Google to find non-published data, case reports and experts’ blogs.

## RESULTS

**Fig. 1.**
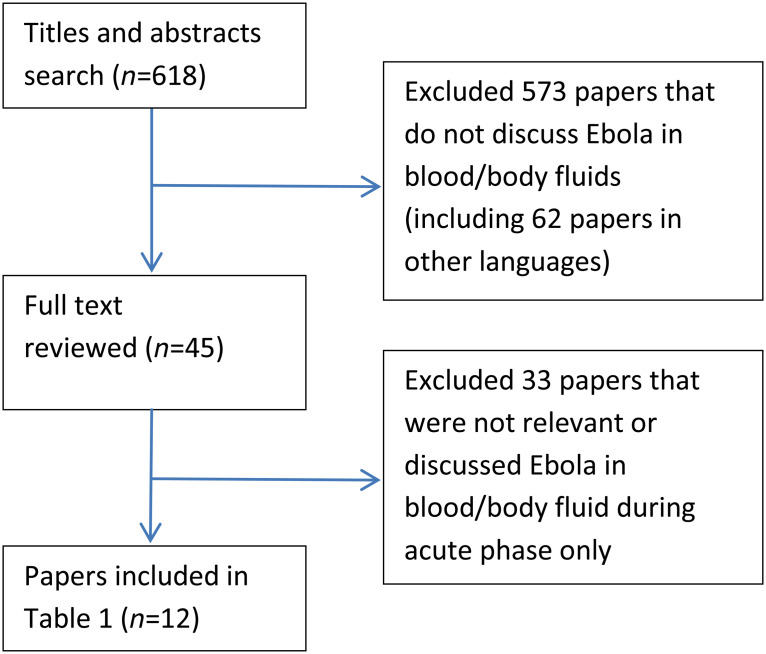
Search strategy and selection of papers.

The initial search yielded 618 papers of which we selected 45 for full text review, in a selection process outlined in Figure 1. We identified 12 studies which examined the presence of EBOV in body fluids of patients who had recovered clinically from the disease. The first evidence of the presence of EBOV in body fluids was provided by Emond and colleagues when a researcher in the UK accidently pricked his thumb while processing material from patients in Africa. EBOV was found in the seminal fluid of this patient, 61 days after the onset of illness. Seminal fluid cultures were repeated on 76, 92 and 110 days after the onset of illness and all were negative for EBOV [6]. Rodriguez *et al.* [4] tested a cohort of 12 convalescent patients during the Kikwit outbreak in 1995 and detected virus RNA in vaginal (day 33), rectal (day 29) and conjunctival (day 22) swabs and in seminal fluid (day 101). Conjunctival, faecal and seminal fluid specimens were negative on days 25, 33 and 700, respectively, while a vaginal fluid specimen was not collected after day 33. Of 11 semen samples collected for culture from convalescent patients, one (9%) was positive for EBOV at day 82 and cultures were not repeated [4]. Rowe *et al.* [7] conducted a prospective cohort study and collected samples from blood, tears, sweat, faeces, urine, saliva, vaginal secretions and semen from 28 convalescent cases. All specimens were tested by ELISA to detect EBOV antigen and cultures were also performed on all specimens to isolate the virus. Semen specimens were also tested by reverse transcriptase–polymerase chain reaction (RT–PCR) to detect viral RNA. EBOV RNA was detected in semen using RT–PCR from days 47 to 91 but was not detected in a sample collected 397 days after of onset of illness. Cultures and antigen test by ELISA on all specimens were negative [7]. A case report of a healthcare worker (HCW) treated in Johannesburg during the Gabon 1995/1996 epidemic showed persistence of viral antigens in urine 23 days after onset of illness, despite being cleared from the blood on day 20. EBOV was also isolated from a semen sample of the index case (day 19) who had assisted the HCW with the placement of a central venous catheter. Follow-up test results of these patients with a definitive negative PCR result were not available [10]. Bausch *et al.* [8] collected acute and convalescent phase samples from a cohort of 26 Ebola patients admitted to Gulua Regional Hospital, in the 2000 epidemic in Uganda. During the acute phase illness, EBOV was cultured from 8% (1/12) of saliva samples and 100% (1/1) from breast milk samples on day 8. Furthermore, 100% of samples of breast milk, tears and blood from epistaxis, 67% (8/12) samples of saliva, 13% (1/8) of sweat and 50% (2/4) of faeces were positive for EBOV RNA by RT–PCR. Culture and RT–PCR were positive for EBOV in a single-test breast-milk sample on day 15 and semen samples (1/2) on day 40 during the convalescent phase. Repeat samples of breast milk and seminal fluid were not collected for culture or RT–PCR [8].

Some studies conducted during the 2014 EBOV outbreak also support the persistence of EBOV in various body fluids. Kreuels *et al.* [5] performed culture on blood, sweat and urine and RT–PCR on sputum, saliva, conjunctival swabs, stool, urine, and sweat of a HCW who contracted disease in Sierra Leone and was treated in Germany. The last culture was positive for EBOV on day 14 in blood and on day 26 in urine, and cultures obtained after day 26 of illness were negative for EBOV. Viral RNA was detected in the urine and sweat until days 30 and 40, respectively [5]. EBOV was detected in urine samples of two US HCWs treated in Emory University Hospital 28 days after onset of illness. A urine sample of the first patient was positive for viral RNA on day 28 and he was discharged on day 30 when two consecutive plasma samples were negative; urine test reports were not provided at discharge. A urine sample of the second patient was negative on day 23 and she was discharged on day 29 of the illness when two consecutive blood samples (collected 24 h apart) were negative [11]. Case reports of Ebola patients treated in the United States also confirmed the presence of viral RNA in various body fluids, including blood, urine, vomitus, faeces, endotracheal secretions and semen; however, the day of illness on which samples were collected was not recorded [12].

Pregnant and lactating women are a special case, with few studies to inform management of the infection risk. Moreau *et al.* [13] reported a case of two lactating mothers from Guinea and discussed mother-to-child transmission through breast milk. The first case was negative for EBOV RNA on day 14 in blood and on day 15 in breast milk, but positive on day 16 in urine; patient was discharged the next day without follow-up test. The child of this mother was infected with EBOV with a known mode of transmission. The second case was negative for EBOV on day 18 but no sample was tested from breast milk [13]. Baggi *et al.* [14] presented a case series of two pregnant women treated at the Médecins Sans Frontières Ebola treatment centre in Guinea. The first case recovered from Ebola and cleared the infection in blood on day 15 of illness (day 8 of admission), and there were persistently high viral loads in the fetal blood, amniotic fluid, placenta and meconium of the newborn on day 22 of illness (day 15 of admission), highlighting the need for infection control precautions in delivery of recovered pregnant women. EBOV RNA was also detected in samples from amniotic fluid and placenta from the second case on day 16 of illness (day 11 of admission), and her blood was negative on day 21 of illness (day 16 of admission); follow-up tests of other specimens were not reported [14].

In March 2015, a 44-year-old Liberian woman died due to EBOV and the mode of transmission in her case is thought to be sexual contact. Her partner had a positive RT–PCR for EBOV in semen (199 days after recovery) and the culture report is yet to come [15]. The WHO declared Liberia free of EBOV transmission in March 2015 and 3 months later a 17-year-old boy died of Ebola. There was no history of travel or contact with other Ebola cases and transmission was likely due to sexual contact [16]. More recently, EBOV was isolated from the aqueous humor of a patient 9 weeks after clinical recovery [17, 18]. Deen *et al*. examined semen samples of 93 male EBOV survivors in Sierra Leone of which 46 (49%) were positive by PCR. Viral RNA was found in semen of survivors 272 days after discharge from hospital and 284 days after onset of symptoms. All survivors had EBOV in the semen at 2–3 months post-onset of EBOV, while 65% and 26% had virus at 4–6 months and at 7–9 months, respectively [19]. Table 1 gives a summary of the studies examining the persistence of EBOV in various body fluids.

We identified only one study that examined the transmission of infection from 29 convalescent patients to 152 households [7]. Baseline and follow-up samples were obtained for 81 household members, none of whom become positive during the convalescent phase of the index case. Five household members had serological evidence of EBOV infection – four were diagnosed as having mild or asymptomatic illness and one was thought to be due to sexual transmission via semen. The sample of the last case was weakly positive for IgM, 52 days after her exposure to the convalescent patient and the semen of patient was positive for EBOV RNA by RT–PCR [7].

Viral RNA has been detected in oral fluid samples from confirmed Ebola cases in the acute phase of illness with 100% sensitivity and specificity [20]. Other studies failed to detect virus in salivary samples during the convalescent phase [4, 5, 7, 8] and it is speculated that salivary enzymes may inactivate the EBOV [8].

## DISCUSSION

Ebola is a poorly studied disease compared to other viruses such as influenza, [21] with a total of 12 studies examining the persistence of the virus in various body fluids during the convalescent phase, with approximately 800 specimens tested in total. The evidence shows that the virus can certainly persist in body fluids after clinical recovery and clearance of the virus from the blood. Beyond this, current evidence is based on a very small number of patients, with variable sampling, and it is important to understand that there will be a range of persistence of virus in different body fluids in convalescence. Certainty about risk of transmission in convalescence cannot be established without larger, statistically robust studies. This is a clear research gap that needs to be filled in order to inform infection control guidelines in convalescence. Some major issues with testing for residual infectiousness of EBOV in various samples are the cost and limited access to level 4 laboratories.

The available studies found that EBOV may persist in semen, aqueous humor, urine and breast milk 82, 63, 26 and 15 days after onset of illness, respectively. Viral RNA is detectable in urine, aqueous humor, sweat, semen, breast milk, faeces and conjunctival fluid, as well as in vaginal secretions from 15 to 272 days. Clearance of the virus from these body fluids may be delayed due to immunological quarantining in those sites [9]. Detection of RNA may or may not indicate the presence of viable infectious virions. As some studies only used PCR, the presence of viable virus cannot be determined. Unless culture is performed using sensitive methods, the significance of a positive PCR with a negative culture result is uncertain; however, given the small number of studies performed with regard to this, infection risk cannot be completely excluded. False-negative viral cultures can occur due to the viral load being too low to be detected in culture media [22] or because of inappropriate specimen handling [4]. EBOV is considered highly infectious and transmission of a small number of viruses could cause clinical illness, which may not be isolated by culture techniques [23].

The mechanism of survival of EBOV in various body fluids and its persistence for a long period is unclear. This may be due to variability in the immune responses of individuals, and within different body compartments, as well as variability in the risk of infection associated with the amount of virus (viral load) an individual is exposed to [9]. It is possible that in some cases, the virus can be partially cleared, corresponding with clinical recovery, but may persist in some tissues beyond this period or relapse at a later time [24]. Some researchers argued that uveitis and arthralgia might be due to separate pathological mechanisms such as direct cytopathic effect and autoimmune reactive arthritis [25]; however, there is a lack of data on the role of immune-mediated phenomena in the sequelae observed in Ebola survivors [26]. There is some evidence that Marburg virus may also persist in seminal fluid up to 2 months after clinical recovery [27, 28]. Marburg virus has been isolated from the aqueous humor of a Marburg patient 2 months after clinical recovery and a repeat viral culture after 10 weeks was negative [29].

If transmission is possible in asymptomatic, convalescent patients, a secondary question is whether transmission can occur from asymptomatically infected subjects. Asymptomatic EBOV infections have been documented [30–32]. In one study during the 1996 outbreak in Gabon, blood was collected from 24 contacts of symptomatic Ebola cases – 46% (11) of whom tested positive for antibodies without symptomatic infection [30]. Another survey showed that 10 (71%) of 14 seropositive (IgG) individuals did not have clinical infection [31]. Reinfection in these asymptomatic cases is controversial [9]. People who recover from Ebola are considered immune to re-infection [33]. However, it is possible that individuals who clear the virus may not have robust enough immunity to survive a challenge with a large viral load, or may actually still be harbouring low levels of virus which may recrudesce [9]. According to a report from Sierra Leone, a patient who survived an initial infection with EBOV was re-infected, presumably due to exposure to a high viral load and a compromised immune system [34]. Similarly a few PCR-negative children who were treated for EBOV in Monrovia, became PCR positive again later [24]. Animal studies also suggest the possibility of reinfection with EBOV [35].

The predominant mode of transmission is direct contact and a significant minority are unable to recall contact with infected people or have no known risk factor [36], raising the possibility of additional infection routes. While direct contact with infectious patients is the main mode of transmission, there is uncertainty around transmission, and likely other modes of transmission [3, 21]. Convalescence may be a period of risk for transmission, but it is poorly studied. Current evidence suggest that blood cultures of Ebola patients become negative after 9–14 days [5, 6, 10, 37] and antigen tests become non-reactive after 15–20 days of onset of illness [5, 10, 37]. While blood is well known to harbour extremely high levels of virus, particularly in the pre-terminal phase where viral loads can be in excess of 10 million copies/ml [38], the role of other body fluids in transmission and the duration of viral persistence capable to producing infection are under-studied.

There is little evidence around transmission during the convalescent phase. More studies are needed to quantify EBOV persistence and infectivity after the acute illness phase is over, and to identify a period in which the virus is cleared from all sites. It is also important in the clinical management of cases to test other fluids for persistence of virus prior to discharge. There is a need for larger studies and more evidence on persistence to inform disease control policy. Given the high case-fatality rate of Ebola and lack of proven, available treatment, all exposure to fluids which are positive by any method should be minimized and HCWs and patients should be informed about the risks. Convalescent patients should be educated about transmission risks and appropriate measures should be taken by their contacts [39]. The small amount of available evidence suggests transmission in convalescence is possible. Active studies to detect EBOV in body fluids during the acute and convalescent phases are needed.

Due to uncertainties around the transmission characteristics, as well as the high mortality rate from infection, it is recommended that patients should be considered potentially infectious for a period of time after immediate clinical recovery [40]. A definite time cannot be given based on the small amount of available evidence, but the longest documented period of persistence can be used as an indicator of infectious potential. Guidelines need to address infection control measures after clinical recovery and during convalescence, when patients could still be infectious. Several studies show the presence of virus in semen and vaginal fluids after recovery [4, 6–8, 19], so sexual intercourse may be avoided for at least 9 months or barrier methods should be used. The WHO recommendation is to abstain from all types of sex or use barrier methods until two consecutive semen samples are negative [41], but this should be updated in light of the findings of new studies [19]. A recent Ebola case in Liberia, likely due to sexual transmission, also called for the adoption of precautionary measures to prevent sexual transmission [42]. The implementation and effectiveness of these measures might be debated due to a lack of data around persistence of EBOV in seminal and vaginal fluids and the duration of infectious period after clinical recovery [43]. Breastfeeding should be avoided during the acute and convalescent phases to prevent vertical transmission [44]. Although there is no evidence of viral shedding in saliva, vomit and sputum during the convalescent phase of illness, further research is required given that only nine small-scale studies have tested this, and none in a consistent way. To account for the normal variation between individuals in virus shedding, large-scale, statistically sound studies are needed of all body fluids which may harbour the virus. Examining the type of EBOV strains in various body fluids is also important, as some strains are associated with high morbidity and mortality.

There are some limitation of this study. Table 1 reports the number of days of EBOV PCR positivity and represents the very last day of detectable viral shedding. Some studies did not explicitly discuss follow-up testing where a definitive negative PCR result was reported. Moreover, we reviewed first, and then moved on to discuss the potential routes of transmission of EBOV for different body fluids, and then the consequences of the prolonged shedding of EBOV in this context for different body fluids and the potential impact/implications for infection control guidelines. Unpublished data is not included in this review. Viable virus has also been isolated from the cerebrospinal fluid of a nurse who presented with neurological symptoms 9 months after clinical recovery [45].

In conclusion, Ebola is an under-studied disease, and there are few studies looking at the persistence of EBOV in body fluids. The few available studies are heterogeneous, show significant variability between patients, do not test the same sites of fluids in a consistent way, and comprise a very small number of total patients tested. Yet even this small number of studies shows that the virus can persist in multiple different fluids after being cleared from the blood. There is a need for more research on persistence, and a uniform approach to infection control guidelines in convalescence. Under these circumstances, given the high case-fatality rate of Ebola and the low infectious dose [46], infection risk in convalescence should be assumed, the precautionary principle applied, and close contacts of convalescent Ebola patients should be given clear guidance on infection control.
